# Combination treatment of RAD001 and BEZ235 exhibits synergistic antitumor activity via down-regulation of p-4E-BP1/Mcl-1 in small cell lung cancer

**DOI:** 10.18632/oncotarget.18984

**Published:** 2017-07-04

**Authors:** Bo Hong, Huogang Wang, Ke Deng, Wei Wang, Haiming Dai, Vivian Wai Yan Lui, Wenchu Lin

**Affiliations:** ^1^ High Magnetic Field Laboratory, Chinese Academy of Sciences, Hefei, Anhui, P. R. China; ^2^ School of Biomedical Sciences, Faculty of Medicine, The Chinese University of Hong Kong, Sha Tin, Hong Kong; ^3^ Anhui Province Key Laboratory of Medical Physics and Technology, Center of Medical Physics and Technology, Hefei Institutes of Physical Science, Chinese Academy of Sciences, Hefei, Anhui, P. R. China

**Keywords:** small cell lung cancer, PI3K/AKT/mTOR, BEZ235, RAD001, p-4E-BP1/Mcl-1

## Abstract

Small cell lung cancer (SCLC) is a highly malignant cancer with few targeted therapies. In the study, by mining the Cancer Cell Line Encyclopedia (CCLE) database, we found that PI3K/AKT/mTOR pathway was aberrant in 92% of SCLC cell lines. Moreover, we found that the phosphorylation level of 4E-BP1 was significantly correlated with SCLC sensitivity to RAD001 (mTOR inhibitor) and BEZ235 (PI3K/mTOR dual inhibitor). Combination of RAD001 and BEZ235 synergistically inhibited the growth of SCLC cells, which was accompanied by enhanced induction of cell cycle arrest and apoptosis. Such a combination dramatically inhibited the activation of AKT, and strongly reduced the phosphorylation of 4E-BP1 and its downstream target Mcl-1. Knock-down of Mcl-1 enhanced the growth inhibition of SCLC cells induced by RAD001 and BEZ235 co-treatment, whereas over-expression of Mcl-1 reduced the growth inhibitory effect. Furthermore, *in vivo* study demonstrated that the combination treatment suppressed tumor growth more effectively than RAD001 or BEZ235 treatment alone. In summary, our study suggests that combination of BEZ235 and RAD001 may be an effective regimen for SCLC treatment, and p-4E-BP1 may serve as a predictive biomarker for SCLC response to mTOR inhibitor.

## INTRODUCTION

Small cell lung cancer (SCLC) is the most aggressive subtype of lung cancer accounting for 10∼15% of the total lung cancer cases. SCLC differs from non-small cell lung cancer (NSCLC) in having neuroendocrine differentiation, higher proliferation index and a tendency to metastasize earlier [1]. At the time of diagnosis, over 70% of SCLC patients are presented with extensive stage disease (SCLC has spread beyond the supraclavicular areas, or with distant metastasis) [2]. Chemotherapy with cisplatin and etoposide is the standard treatment for extensive stage SCLC, and the initial response rate is high. However, the major issue is that almost all patients relapse within 3–6 months. Therefore, extensive stage SCLC has an extremely very poor prognosis with overall 5-year survival rate of 1%–2%. Thus, effective new therapy for advanced SCLC patients is needed [3, 4].

Previous studies have identified PI3K/AKT/mTOR pathway as a promising target for SCLC. Genomic analysis of 51 SCLC patient specimens has revealed that about 36% of the SCLC tumors harbor genetic alterations in the PI3K/AKT/mTOR pathway, including point mutations and copy number changes in *AKT2*, *AKT3*, *PTEN*, *PIK3CA*, *RICTOR* and *MTOR* [5]. Marinov et al. found that mTOR protein and its downstream targets were also up-regulated in human SCLC cell lines and patient specimens. Moreover, the mTOR inhibitor, RAD001, can decrease the growth of SCLC cells *in vitro* and *in vivo* [6]. However, clinical trials indicated that RAD001 had limited activity in SCLC as a monotherapy. In a phase II trial of RAD001 in 35 patients with relapsed SCLC, the results reported that only one patient had a partial response, 8 had stable disease, and 26 had disease progression [7]. Thus, new therapeutic strategies need to be developed to improve the efficacy of RAD001 in SCLC.

mTOR is a key serine/threonine protein kinase that regulates cellular growth, proliferation and survival via mTOR complex 1 (mTORC1) and mTOR complex 2 (mTORC2) [8]. RAD001, a rapamycin derivative, suppresses cancer cell growth through inhibiting mTORC1 and its downstream targets 4E-BP1 and S6 kinase. However, in some cell context, RAD001 is not able to completely inhibit the phosphorylation of 4E-BP1 [9, 10]. Moreover, AKT could be activated by RAD001 through the blockade of the S6K-mediated negative feedback loop [11, 12]. Therefore, incomplete inhibition of 4E-BP1 phosphorylation and AKT feedback activation are thought to contribute to the resistance of cancer cells to RAD001 treatment [13]. BEZ235 (a PI3K, mTORC1 and mTORC2 inhibitor) is able to effectively inhibit the activation of 4E-BP1 and AKT [14]. Previous studies have demonstrated that BEZ235 exerts synergistic anti-tumor activities when combined with RAD001 in various tumor models, including NSCLC, glioma, renal cancer, pancreatic cancer and breast cancer [15–17]. Thus, we hypothesized that antitumor efficacy of RAD001 could be enhanced when combined with BEZ235 in SCLC.

In the study, we initially demonstrated that PI3K/AKT/mTOR pathway was aberrant in SCLC cell lines by mining the Cancer Cell Line Encyclopedia (CCLE) database [18]. We found that basal level of p-4E-BP1 was significantly correlated with the sensitivity of SCLC cells to RAD001 and BEZ235. Importantly, our study investigated that combination treatment with RAD001 and BEZ235 synergistically inhibited growth of SCLC cells through down-regulation of p-4E-BP1 and its downstream target Mcl-1.

## RESULTS

### PI3K/AKT/mTOR pathway is active in SCLC cell lines

**Figure 1:**
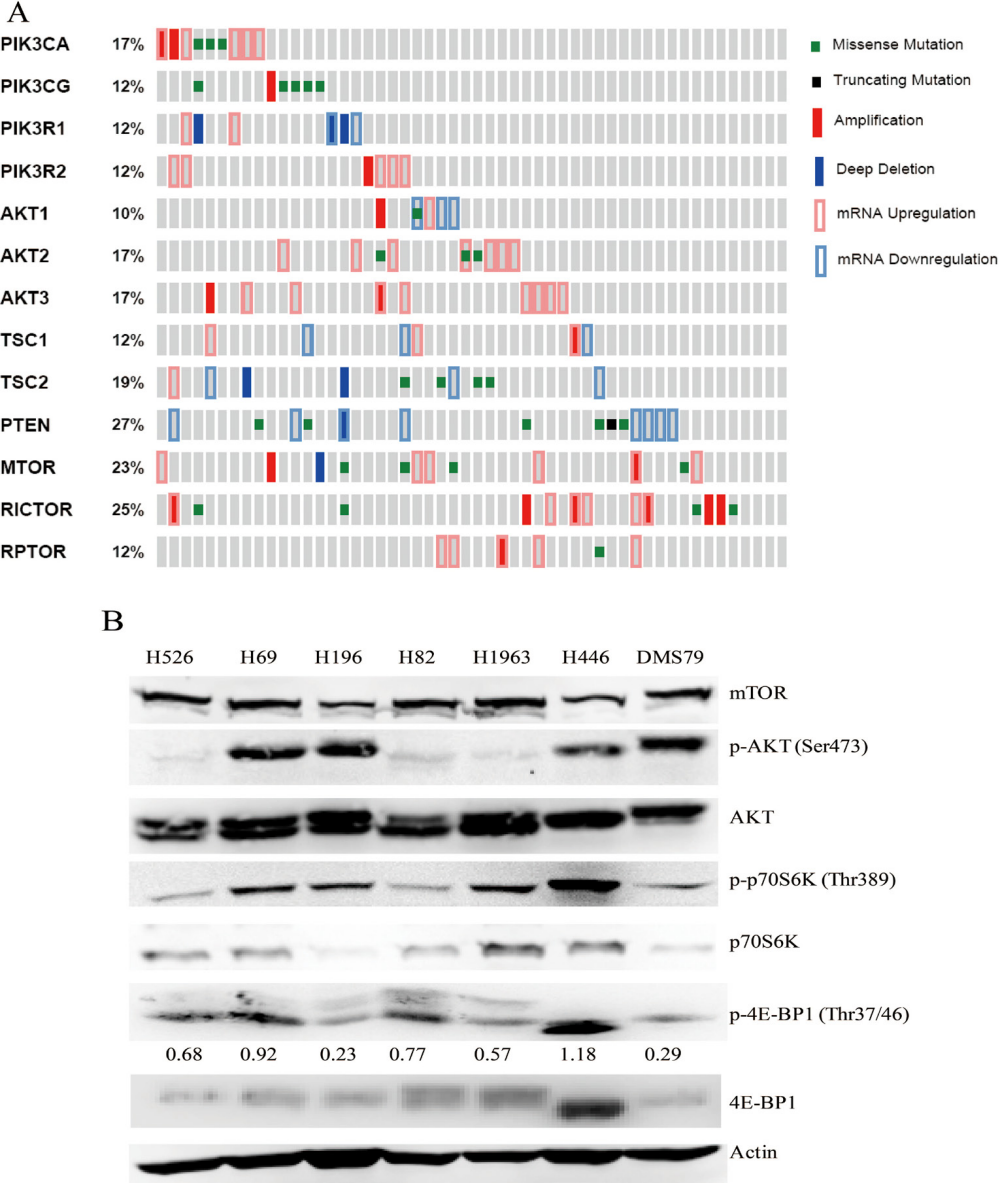
(**A**) OncoPrinter shows the distribution and frequency of somatic mutations, copy number changes and mRNA expression levels of the genes involved in PI3K/AKT/mTOR pathway in SCLC cell lines. Data was generated by CCLE and obtained via the cBioPortal for Cancer Genomics (URL: http://www.cbioportal.org/public-portal/). Gray bars represent individual SCLC cell lines. (**B**) Protein expression and phosphorylation of the key kinases of PI3K/AKT/mTOR pathway in 7 SCLC cell lines by western blot analysis. Actin was used as a loading control. The density of the bands of p-4E-BP1 was quantified and normalized to Actin.

Previous study has indicated that 36% of SCLC patients’ tumor samples harbor genetic alterations in PI3K/AKT/mTOR pathway [5]. However, no study to date has reported genetic characterization of PI3K/AKT/mTOR pathway in the SCLC cell line panel. In this study, by mining the CCLE database [18], we comprehensively analyzed the genomic aberrations of the PI3K/AKT/mTOR pathway in a panel of 52 SCLC cell lines. The analysis included mutations and copy number changes, as well as mRNA expression levels of key molecules of the PI3K/AKT/mTOR pathway. The OncoPrinter plot indicated that 92% of SCLC cell lines (48/52) harbor point mutations, copy number changes and abnormal gene expression in the key genes of PI3K/AKT/mTOR pathway (Figure 1A). The mutation sites of these genes were mapped onto their protein domains (Supplementary Figure 1). Recurrent mutations reported by COSMIC and oncogenic mutations confirmed by previous study were marked in the MutationMapper (Supplementary Figure 1). These results indicate that PI3K/AKT/mTOR signaling is aberrant in SCLC cell lines.

Next, we detected the activation status of PI3K/AKT/mTOR pathway in SCLC cell lines. As shown in Figure 1B, mTOR protein was constitutively expressed in all tested SCLC cell lines (H526, H82, DMS79, H69, H1963, H196 and H446). All SCLC cell lines examined displayed phosphorylation of p70S6K and 4E-BP1, although the degree of phosphorylation varied among them. Phosphorylation of AKT was more strongly expressed in H69, H196, H446 and DMS79 cells compared with H526, H82 and H1963 cells. Among all of SCLC cell lines tested, H446 cells exhibited the strongest activation of mTOR signaling, as indicated by the highest expression of phosphorylated p70S6K and 4E-BP1. In all tested SCLC cell lines, the activation status of PI3K/AKT/mTOR pathway is consistent with molecular genetic data, which shows that H446 cell line harbors the most alterations in the key genes of PI3K/AKT/mTOR pathway, and other cell lines harbor at least one alteration in the pathway (Table 1). Taken together, these data suggest that PI3K/AKT/mTOR pathway is active in SCLC cell lines.

**Table 1:** Genetic alterations of the PI3K/AKT/mTOR pathway in SCLC cell lines analyzed in this study

	H526	H69	H196	H82	H1963	H446	DMS79
PIK3CA		G106_R108del					
PIK3CG	P563T				S806F		
PIK3R1		UP					
PIK3R2				AMP		UP	
AKT2	UP						
AKT3			UP		UP	UP	
TSC1						DOWN	
TSC2						E75K	
PTEN			Y138C		DOWN	DOWN	DOWN
MTOR						M2345V	
RICTOR			AMP				

UP: mRNA level up, DOWN: mRNA level down, AMP: copy number amplification.

### Basal level of phosphorylated 4E-BP1 is significantly correlated with the sensitivity of SCLC cells to RAD001 and BEZ235

**Figure 2:**
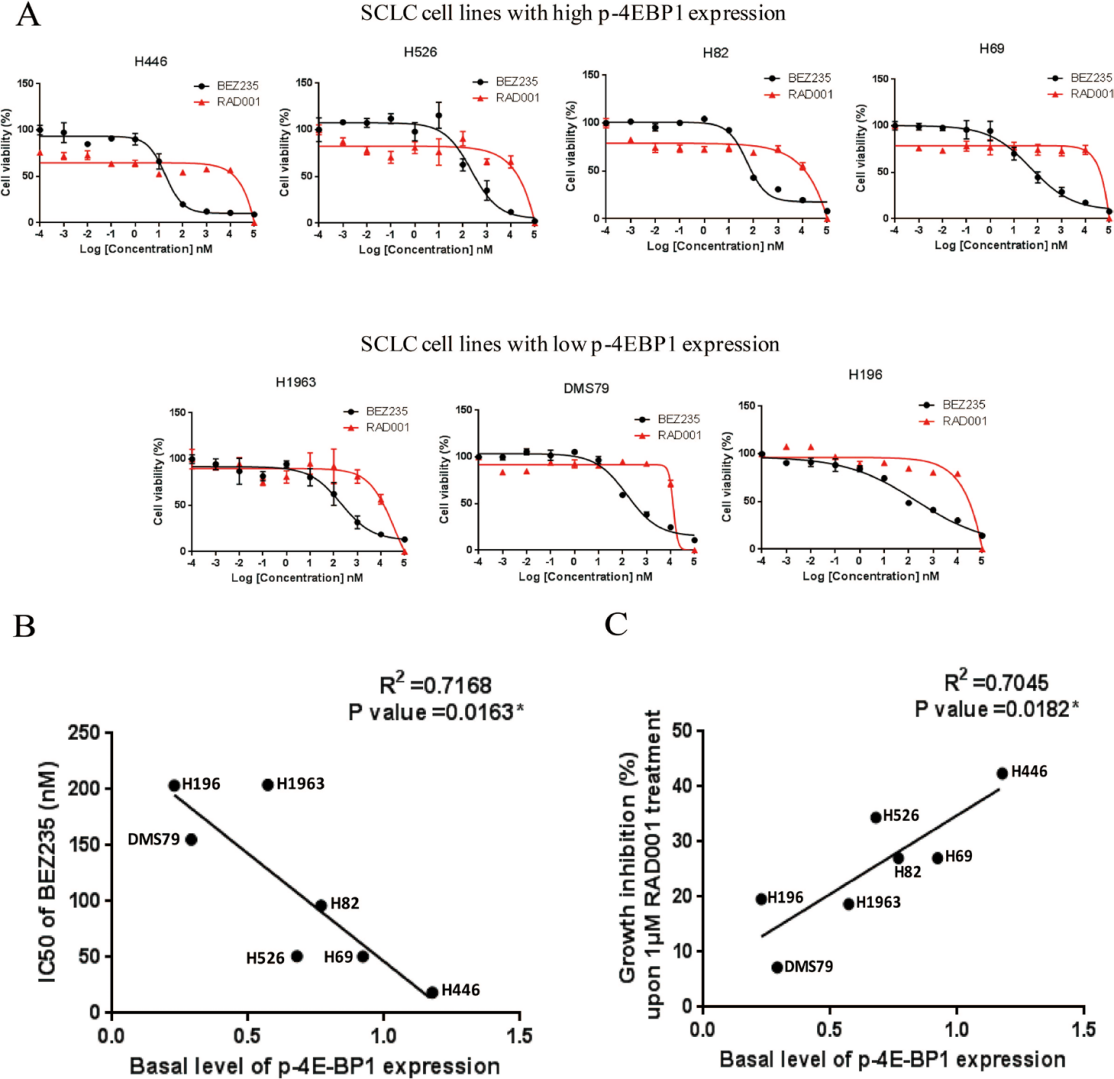
(**A**) Growth inhibition curves of RAD001 and BEZ235 in a panel of SCLC cell lines. SCLC cells were treated with different concentrations of RAD001 or BEZ235 for 72 hr. CellTiter-Glo Luminescent assay was performed to evaluate the cell proliferation. (**B**) Basal level of phosphorylated 4E-BP1 is significantly correlated with the IC50 of BEZ235 in SCLC cell lines. Basal levels of phosphorylated 4E-BP1 in SCLC cell lines were quantified on the western blot (Figure 1B). IC50s of BEZ235 were obtained from the growth inhibition curves of BEZ235 (Figure 2A). (**C**) Basal level of phosphorylated 4E-BP1 significantly is correlated with the growth inhibition of SCLC cells upon 1 μM RAD001 treatment. The percentage of growth inhibition of SCLC cells upon 1 μM RAD001 treatment was obtained from the growth inhibition curves of RAD001 (Figure 2A).

Since PI3K/AKT/mTOR pathway is active in SCLC cell lines, we further examined the anti-tumor activity of RAD001 and BEZ235 in a panel of SCLC cell lines. As shown in Figure 2A, BEZ235 induced a marked growth inhibition in all of SCLC cell lines with IC50 values in the nanomolar range (Table 2). However, SCLC cells were rather insensitive to RAD001 with IC50 values of over 10 μM, as 10 μM RAD001 treatment only inhibited the growth of SCLC cells by 20.7–45.0% (Figure 2A). Interestingly, RAD001 was able to inhibit 12.8%–36.1% of cell growth at very low concentrations (0.001 nM–1 nM) in some SCLC lines with relatively higher endogenous levels of phosphorylated 4E-BP1 (Figure 2A).

**Table 2:** Correlation between cell sensitivity to BEZ235 (or RAD001) and basal level of p-4E-BP1

	SCLC cell lines						
	Sensitive to BEZ235 and RAD001				Insensitive to BEZ235 and RAD001		
Parameters	H446	H526	H69	H82	DMS79	H1963	H196
IC50 (nM) of BEZ235	18.4	51.0	50.7	96.1	155.3	204.1	203.4
Growth inhibition (%) upon 1 μM RAD001 treatment	42.4	34.4	27.0	27.0	7.2	18.7	19.6
Basal level of p-4E-BP1	1.18	0.68	0.92	0.77	0.29	0.57	0.23

We then examined the relationship between basal level of phosphorylated 4E-BP1 and SCLC sensitivity to RAD001 and BEZ235 (Table 2). Basal level of phosphorylated 4E-BP1 in SCLC cell lines was quantified by densitometry in Figure 1B. As shown in Figure 2B and 2C, the level of phosphorylated 4E-BP1 was significantly correlated with the IC50 of BEZ235, and the correlation between the level of phosphorylated 4E-BP1 and the percentage of growth inhibition of SCLC cells upon 1 μM RAD001 treatment was also significant. H446 cells, which expressed the highest level of p-4E-BP1, were the most sensitive to BEZ235 and RAD001 among all SCLC cell lines tested. Taken together, these data suggest that p-4E-BP may be mechanistically associated with the anti-tumor response of BEZ235 and RAD001 in SCLC.

### Combination treatment of BEZ235 and RAD001 shows synergistic antitumor effect in SCLC

**Figure 3:**
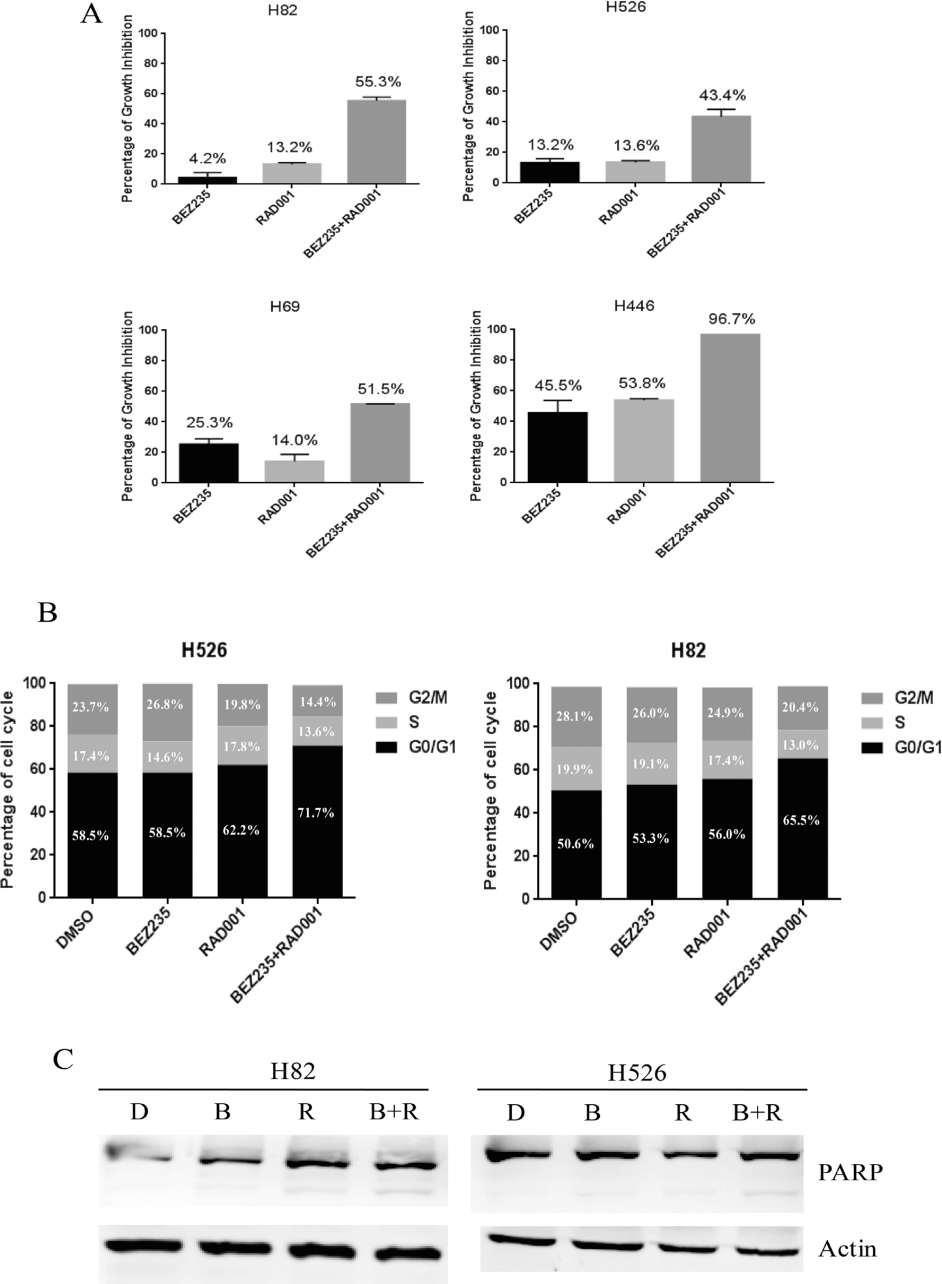
(**A**) Dramatic growth inhibition of SCLC cells induced by co-treatment of RAD001 and BEZ235. SCLC cells were treated with DMSO control, RAD001 (10 nM), BEZ235 (10 nM) or the combination of RAD001 (10 nM) and BEZ235 (10 nM) for 72 hr. Growth inhibition was determined by CellTiter-Glo Luminescent assay after treatment. (**B**) Cell cycle distributions of H526 and H82 cells treated with DMSO control, RAD001 (10 nM), BEZ235 (10 nM) or the combination of RAD001 (10 nM) and BEZ235 (10 nM) for 24 hr. (**C**) PARP cleavage induction of H526 and H82 cells treated with DMSO control, RAD001 (10 nM), BEZ235 (10 nM) or the combination of RAD001 (10 nM) and BEZ235 (10 nM) for 24 hr. Full-length, cleaved PARP, and loading control Actin were detected by western blot. D: DMSO, B: BEZ235, R: RAD001, B+R: BEZ235+RAD001.

Given the insensitivity of SCLC cells to RAD001 treatment alone, we next examined if the efficacy of RAD001 could be potentially enhanced when combined with BEZ235. SCLC cells were treated with DMSO control, RAD001 (10 nM), BEZ235 (10 nM) and the combination of RAD001 (10 nM) and BEZ235 (10 nM) for 72 hr. Cell proliferation assay indicated that co-treatment with BEZ235 and RAD001 remarkably inhibited SCLC cell growth when compared with the single agents alone (Figure 3A). As shown in Table 3, the combination indexes (CI) of all SCLC cell lines examined were less than 1, indicating synergistic inhibitory effect on SCLC cell growth. Furthermore, the combination indexes (CI) in H82, H526, H1963, H196 and H446 cell lines were less than 0.1, indicating very strong synergism, followed by DMS79 and H69 with CI values between 0.1 and 0.3 for strong synergism.

**Table 3:** Combination index (CI) of BEZ235 and RAD001 in SCLC cell lines

Cell lines	BEZ235 dose *D1* (nM)	RAD001 dose *D2* (nM)	Growth inhibition x (%)	Dose of BEZ235 alone with same inhibition *(Dx)1* (nM)	Dose of RAD001 alone with same inhibition *(Dx)2* (nM)	CI
H82	10	10	55.3	107.2	19054.6	0.09^**^
H526	10	10	43.4	213.8	14454.4	0.05^**^
DMS79	10	10	34.7	100.0	11749.0	0.10^*^
H69	10	10	51.5	75.9	50118.7	0.13^*^
H1963	10	10	39.9	151.4	7244.4	0.07^**^
H196	10	10	52.9	323.6	21877.6	0.03^**^
H446	10	10	96.7	10000.0	10000.0	< 0.01^**^

^**^Very strong synergism, ^*^Strong synergism.

We next analyzed the effect of RAD001 and BEZ235 on cell cycle and apoptosis. Upon single treatment of H82 and H526 cells with 10 nM of RAD001 or 10 nM of BEZ235 for 24 hr, we detected an increasing proportion of cells in the G1 phase when compared with DMSO treatment, and combination treatment had an additional effect on G1 phase arrest (Figure 3B and Supplementary Figure 2). We also evaluated the potential induction of apoptosis by PARP cleavage when H82 and H526 cells were treated with the single agents alone or in combination. As shown in Figure 3C, the combined treatment enhanced apoptosis as PARP cleavage was increased compared with BEZ235 or RAD001 single treatment (Figure 3C). The results indicate that combination treatment of RAD001 and BEZ235 synergistically inhibited the growth of SCLC cells, which was accompanied by enhanced induction of cell cycle arrest and apoptosis.

### Combination treatment with BEZ235 and RAD001 dramatically suppresses PI3K/AKT/mTOR signaling in SCLC cells

**Figure 4:**
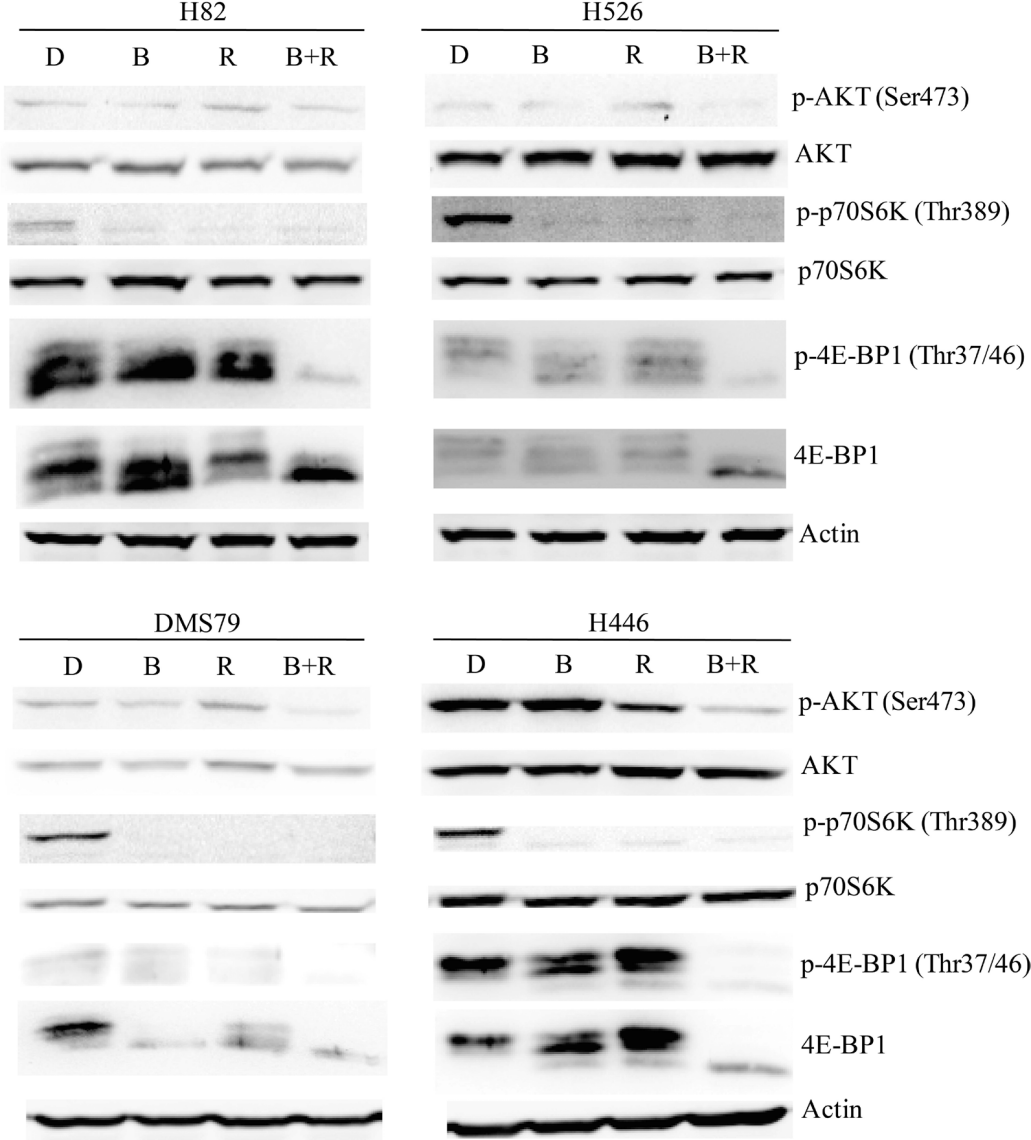
Remarkable inhibition of PI3K/AKT/mTOR signaling of SCLC cells by RAD001 and BEZ235 co-treatment SCLC cells (H82, H526, DMS79 and H446) were treated with DMSO control, RAD001 (10 nM), BEZ235 (10 nM) or the combination of RAD001 (10 nM) and BEZ235 (10 nM) for 12 hr. Cellular proteins were collected for western blot. D: DMSO, B: BEZ235, R: RAD001, B+R: BEZ235+RAD001.

To investigate the molecular mechanism(s) underlying the synergistic effect of the BEZ235/RAD001 combination in SCLC cells, western blot was performed to examine several potential targets of the PI3K and mTOR pathway. As shown in Figure 4, 10 nM RAD001 treatment alone was not able to completely block the activation of 4E-BP1, a downstream effector of mTOR, even though the dose of RAD001 was increased to 100 nM (Supplementary Figure 3). While RAD001 completely inhibited the phosphorylation of p70S6K in H82, H526, DMS79 and H446 cells (Figure 4). Furthermore, RAD001 (both 10 nM and 100 nM) treatment increased p-AKT in H526 and DMS79 cells (Figure 4 and Supplementary Figure 3). These data are consistent with previous findings that cancer cells are resistant to RAD001 due to incomplete inhibition of p-4E-BP1 and AKT feedback activation [13]. Importantly, the combination of BEZ235 (10 nM) and RAD001 (10 nM) effectively blocked PI3K/AKT/mTOR signaling in all tested SCLC cell lines, as indicated by almost complete blockade of p-4E-BP1, p-p70S6K and p-AKT expression in all SCLC cell lines tested (Figure 4). Our data demonstrated that the combination of RAD001 and BEZ235 remarkablely inhibited 4E-BP1 and AKT activation, which likely contribute to the synergistic inhibitory activity observed.

### Anti-apoptotic protein Mcl-1 modulates the synergistic anti-tumor effect of BEZ235 and RAD001 in SCLC cells

**Figure 5:**
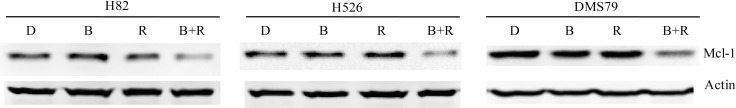
Remarkable inhibition of Mcl-1 protein expression in SCLC cells co-treated by RAD001 and BEZ235 SCLC cells were treated with DMSO control, RAD001 (10 nM), BEZ235 (10 nM) or the combination of RAD001 (10 nM) and BEZ235 (10 nM) for 12 hr. Cellular proteins were collected for western blot. Actin was used as the loading control. D: DMSO, B: BEZ235, R: RAD001, B+R: BEZ235+RAD001.

**Figure 6:**
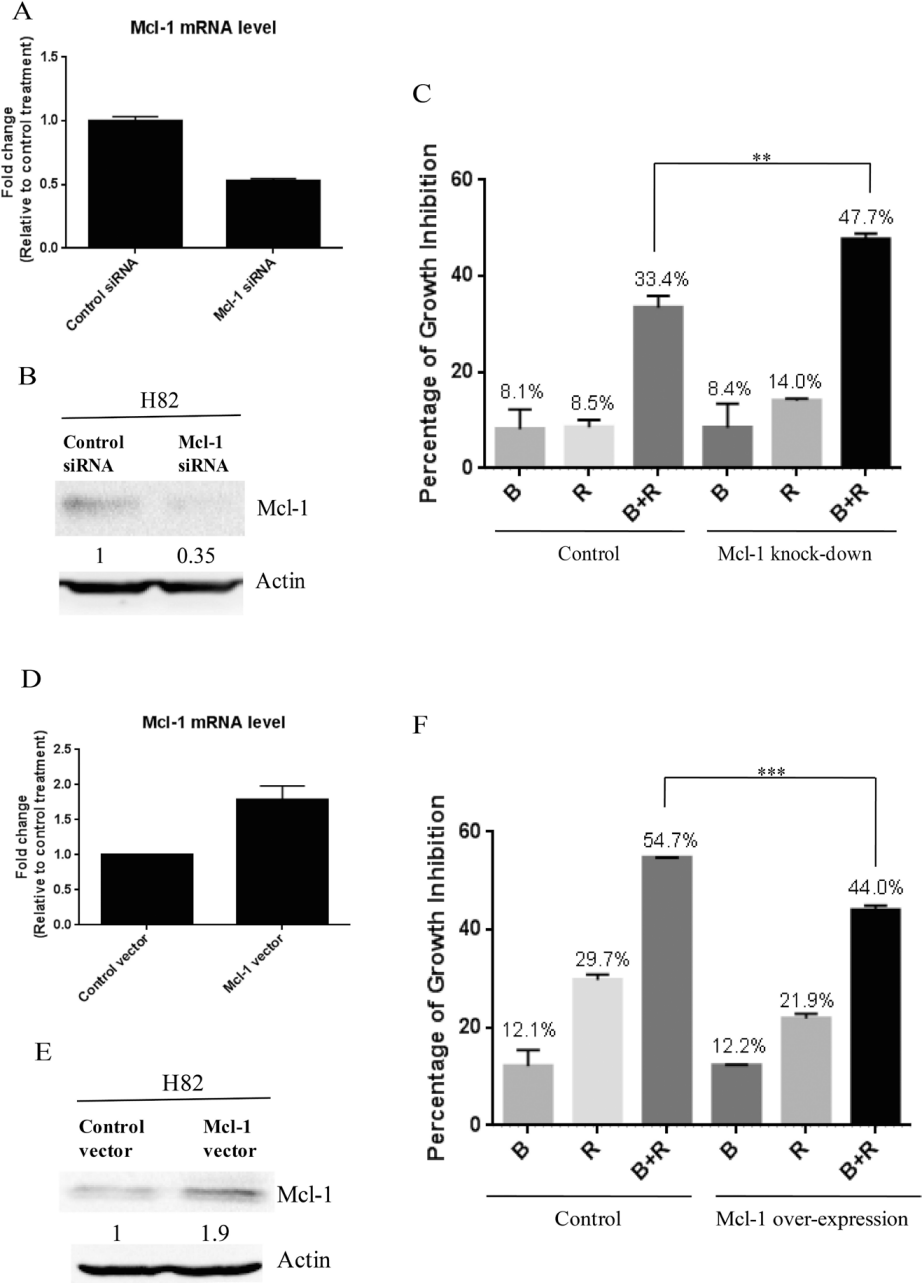
(**A**) Mcl-1 knock-down confirmed by qRT-PCR. After H82 cells were transfected with Mcl-1 siRNA or control siRNA for 48 hr, total RNA were collected. Mcl-1 mRNA was detected by qRT-PCR. (**B**) Mcl-1 knock-down confirmed by western blot. (**C**) Mcl-1 knock-down enhances the growth inhibition of SCLC cells induced by BEZ235 and RAD001 co-treatment. After H82 cells were transiently transfected with Mcl-1 or control siRNA for 24 hr, transfected cells were treated with DMSO, RAD001 (10 nM), BEZ235 (10 nM) or the combination of RAD001 (10 nM) and BEZ235 (10 nM) for 72 hr. Cell proliferation was evaluated by CellTiter-Glo Luminescent assay. (**D**) Mcl-1 over-expression confirmed by qRT-PCR. (**E**) Mcl-1 over-expression confirmed by western blot. (**F**) Mcl-1 over-expression reduces the growth inhibition of SCLC cells induced by BEZ235 and RAD001 co-treatment.

Previous study has demonstrated that the growth inhibition of cancer cells by mTOR inhibitor treatment was p-4E-BP1 dependent [19], which is consistent with our finding. 4E-BP1 phosphorylation is known to enable the translation initiation of Mcl-1, a critical protein for cancer cell survival [20]. After combination treatment of BEZ235 and RAD001, we observed a marked reduction of 4E-BP1 phosphorylation in SCLC cells (Figure 4). Thus, we speculated that co-treatment of BEZ235 and RAD001 could inhibit the expression of Mcl-1 through the down-regulation of p-4E-BP1. We did detect the dramatic reduction of Mcl-1, when H82, H526 and DMS79 cells were co-treated with BEZ235 and RAD001 for 12 hr (Figure 5). To show whether Mcl-1 modulates the growth inhibition by BEZ235 and RAD001 co-treatment in SCLC cells, we knocked down or over-expressed Mcl-1 transiently in H82 cells by transfecting Mcl-1 siRNA or expression plasmid. Knock-down or over-expression of Mcl-1 was validated by qRT-PCR and western blot (Figure 6A, 6B, 6D and 6E). Cell proliferation assay demonstrated that combination treatment of BEZ235 and RAD001 induced more growth inhibition when H82 cells were transfected with Mcl-1siRNA, compared with control siRNA transfection (Figure 6C). However, when Mcl-1 was over-expressed in H82 cells, the growth inhibitory effect was reduced (Figure 6F). These data indicate that Mcl-1 is involved in modulating the growth inhibition of SCLC cells treated by BEZ235 and RAD001 combination.

### BEZ235/RAD001 combination remarkablely inhibits SCLC tumor growth *in vivo*

**Figure 7:**
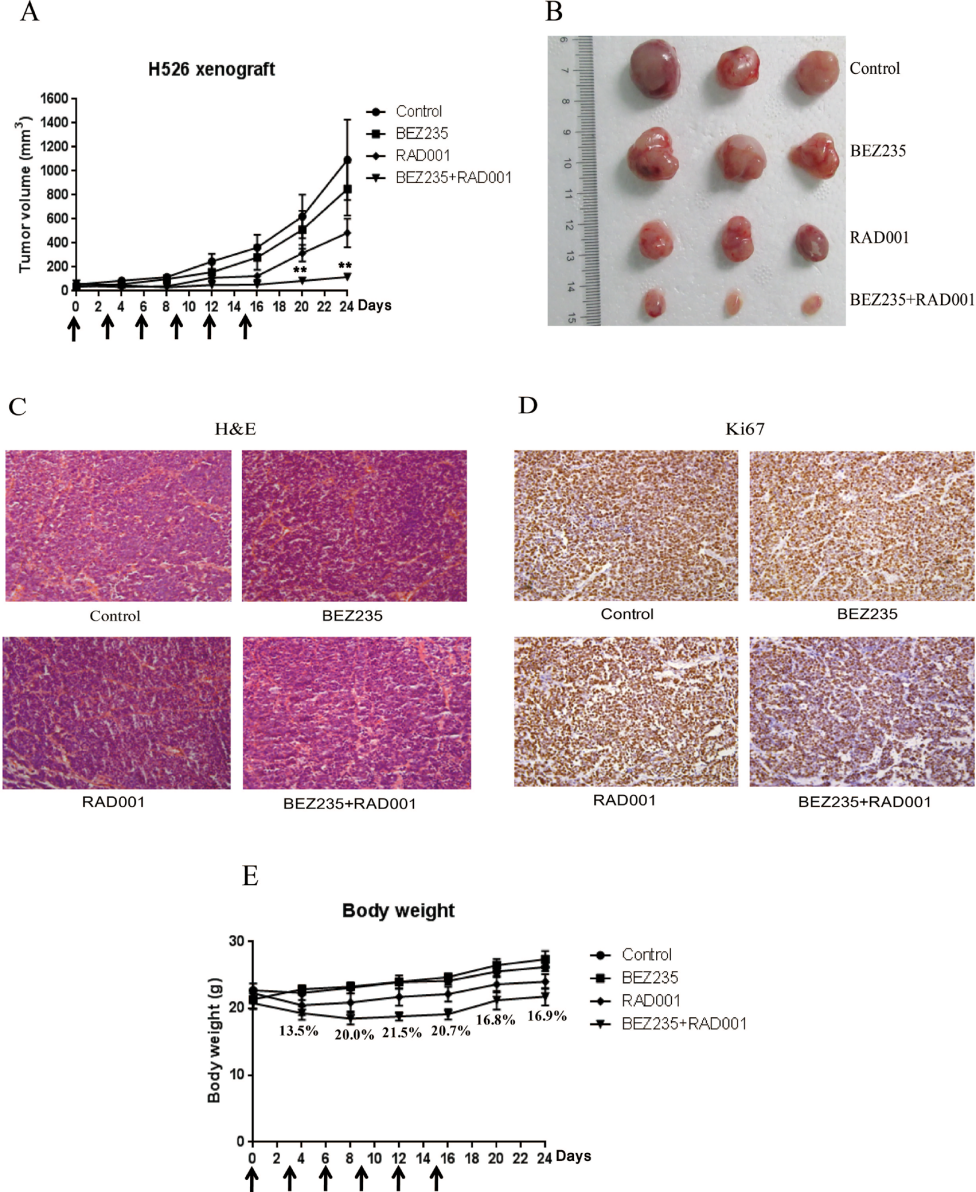
(**A**) Combination treatment with BEZ235 and RAD001 significantly induced growth inhibition of SCLC xenograft. H526 xenografts were treated by DMSO control, 2.5 mg/kg BEZ235, 2.5 mg/kg RAD001, and the combination of BEZ235 (2.5 mg/kg) and RAD001 (2.5 mg/kg) every three days. The arrows show the days of the treatment. The mean tumor size ± SEM is shown (^**^P < 0.01 by an unpaired *t* test). (**B**) Imaging of representative tumors from each group. The tumors were excised at the end of the experiment. (**C**) H&E staining. (**D**) Immunohistochemical detection of Ki67. (**E**) Body weights of the mice during the course of treatment. The mean body weight ± SEM is shown. The numbers represent the percentage of body weight loss in the combination group compared with control group.

Because of the strong *in vitro* synergistic anti-tumor activity of BEZ235 and RAD001 combination in SCLC, we further tested the therapeutic combination of BEZ235 and RAD001 *in vivo*. Mice bearing xenografted H526 SCLC tumors were intraperitoneally treated with vehicle control, BEZ235, RAD001, and the combination of BEZ235 and RAD001 every three days for 15 days. As shown in Figure 7A and 7B, combination treatment with BEZ235 and RAD001 significantly inhibited tumor growth of H526 xenografts as compared with vehicle-treated control. Both RAD001 and BEZ235 treatment alone partially, but not significantly, inhibited the growth of H526 xenografts. Histological analyses showed that co-treatment with BEZ235 and RAD001 strongly decreased the expression of proliferation marker Ki-67 (Figure 7C and 7D). It should be noted that the combination treatment caused ∼20% loss of body weight after treatment. But mice were tolerant to the combination treatment, as all treated mice survived and the body weight increased rapidly after release of the treatment (Figure 7E).

## DISCUSSION

In present study, we reported that PI3K/AKT/mTOR pathway was active in an array of SCLC cell lines. SCLC cells with higher expression of p-4E-BP1 were more sensitive to RAD001 and BEZ235. Moreover, our study demonstrated that RAD001 exerted a synergistic antitumor effect when combined with BEZ235 in SCLC, via p-4E-BP1/Mcl-1 down-regulation.

Our study found that RAD001 and BEZ235 inhibited SCLC cell growth in a different manner (Figure 2A). In SCLC cell lines with higher p-4E-BP1 expression, RAD001 at very low concentration (0.001 nM–1 nM) was able to moderately inhibit SCLC cell growth by 12.8%–36.1%, whereas the growth inhibition was not enhanced as RAD001 concentrations’ increase unless RAD001 reached very high concentrations (100 μM). Unlike RAD001, BEZ235 inhibited SCLC cell growth in a dose-dependent manner. The differential response of SCLC cells to RAD001 and BEZ235 may be due to different drugs’ actions. RAD001, a rapamycin analogue, is a specific allosteric mTORC1 inhibitor. By binding to mTORC1 through its association with FKBP12, RAD001 prevents mTORC1 from interacting with its substrates (S6K and 4E-BP1), thereby inhibiting the activation of mTORC1 substrates [21]. BEZ235 is an ATP competitive inhibitor that potently reduces the kinase activity of PI3K, mTORC1 and mTORC2 [22]. The differential drug actions of RAD001 and BEZ235 suggest that it could be feasible to treat cancer cells with this combination, although they both target mTOR.

Our study found that SCLC cells were not sensitive to RAD001, which is likely due to incomplete inhibition of 4E-BP1 phosphorylation. Previous studies have demonstrated that mTOR inhibitor-induced growth inhibition correlated with the degree of p-4E-BP1 inhibition. Ducker et al. reported that if p-4E-BP1 can be effectively inhibited by the mTOR inhibitor PP242, the colon cancer cells and patient-derived xenografts were PP242 sensitive; On the contrary, in PP242-resistant colon cancer cells and patient-derived xenografts, p-4E-BP1 was poorly inhibited by PP242 [19]. Nishikawa et al. reported that the growth inhibitory effect of mTOR inhibitors temsirolimus and Ku-0063794 was closely associated with the degree of p-4E-BP1 inhibition in bladder cancer [23]. Similarly, our study found that growth inhibitory efficacy of BEZ235, RAD001 and their combination in SCLC cells were closely associated with the degree of p-4E-BP1 inhibition. Furthermore, we found that BEZ235/RAD001 combination inhibited SCLC growth through the 4E-BP1 downstream effector, Mcl-1. Therefore, our study indicates that mTOR/4E-BP1/Mcl-1 axis is important for the survival of SCLC cells, and targeting of this key signaling axis can be effective against SCLC.

Our study indicated that basal level of p-4E-BP1 significantly correlated with the SCLC sensitivity to RAD001 and BEZ235. Previous studies have reported an association between mTOR inhibitor sensitivity and the basal level of p-4E-BP1 in cancer cells. In gastric cancer, Nishi et al. reported that RAD001-sensitive cells significantly expressed higher level of p-4E-BP1 compared with RAD001-resistant cells [24]. In endometrial cancer, Darb-Esfahani et al. reported that Ishikawa cells with higher level of p-4E-BP1 is more sensitive to mTOR inhibitor rapamycin, compared with HEC-1A cells with lower level of p-4E-BP1 [25]. Taken together, our and other studies suggest that p-4E-BP1 may be an effective biomarker to predict mTOR inhibitor sensitivity in SCLC as well as in other cancers. The evaluation of p-4E-BP1 expression in tumor tissues may help patient selection for personalized treatment with mTOR inhibitor to improve clinical outcome.

In conclusion, our study demonstrates that combination of BEZ235 and RAD001 synergistically inhibits the growth of SCLC cells through down-regulation of p-4EBP-1/Mcl-1. Phosphorylated 4E-BP1 is a potential predictive biomarker for the efficacy of mTOR inhibitors in SCLC cells. Results from our study warrant further clinical investigation of BEZ235/RAD001 combination for SCLC personalized therapy with p-4E-BP1 as a biomarker for patient selection.

## MATERIALS AND METHODS

### Materials

RAD001 and BEZ235 were purchased from Selleck chemical, Shanghai, China, and stock solutions were prepared in DMSO (Sigma–Aldrich, Saint Louis, MO, USA) at a concentration of 10 mM and 2 mM, respectively. Antibodies against phospho-AKT, total AKT, phospho-p70S6K, total p70S6K, phospho-4E-BP1, total 4E-BP1, PARP, mTOR and Mcl-1 were from Cell Signaling Technology, Danvers, MA, USA. Actin antibody was from TransBionovo, Beijing, China. The siRNA against human Mcl-1 as well as its negative control siRNA were purchased from Cell Signaling Technology (Danvers, MA, USA). Mcl-1 expression vector (pSPN-Mcl-1) and control vector (pSPN) were kindly provided by professor Haiming Dai (Chinese Academy of Sciences, Hefei, China).

### Cell lines and cell culture

The human SCLC cell lines, H82, H526, DMS79, H69, H1963, H446 and H196 were maintained in RPMI-1640 media supplemented with 10% fetal bovine serum (FBS) and 1% penicillin/streptomycin in a humidified incubator at 37°C in 5% CO_2_. All of these SCLC cell lines are kindly provided by Dr. Matthew Meyerson at Dana-Farber Cancer Institute, USA. RPMI-1640 media, FBS and penicillin/streptomycin were purchased from Gibco, Life Technologies, Carlsbad, CA, USA.

### Cell proliferation assay

SCLC cells (3000 cells per well) were treated with drugs or vehicle control (DMSO) for 72 hr. Cell viability was determined by CellTiter-Glo Luminescent assay (Promega, Madison, WI, USA). Percentage of cell viability (mean ± SEM) at each dose was calculated against the respective DMSO control. The IC50 values were determined from the sigmoidal dose–response curves using PRISM4 software (GraphPad Software, Inc., La Jolla, CA, USA).

### Cell cycle analysis

SCLC cells were treated with DMSO, RAD001 (10 nM), BEZ235 (10 nM) and the combination of RAD001 (10 nM) and BEZ235 (10 nM) for 24 hr. After treatment, the cells were fixed in 70% cold ethanol and incubated at −20°C overnight, and then stained with PI/RNase staining buffer (BD Pharmingen, San Diago, CA, USA). Flow cytometry was performed using a FACS Calibur (BD Pharmingen), and results were analyzed by ModFit software (Verity Software House).

### Western blot

After drug treatment, protein lysates were collected for western blot analysis as previously described [26]. Twenty-five micrograms of protein was used for SDS-PAGE. After primary and secondary antibody incubations, the signal was detected with the Supersignal West Pico Chemiluminescent detection kit (Thermo Fisher Scientific, Waltham, MA, USA), followed by autoradiography.

### Quantitative RT-PCR (qRT-PCR)

Total RNA was isolated using TransZol Up Plus RNA Kit (Trans GeneBiotech, Beijing, China). cDNA was synthesized by QuantiNova Reverse Transcription Kit (QIAGEN, Hilden, Germany) according to the manufacturer’s instructions. The quantitative real-time PCR was performed in triplicate using a FastStart Essential DNA Green Master (Roche, Mannheim, Germany) on a Roche LightCycler 96 Real Time PCR System. The expression level of Mcl-1 was normalized to the expression level of β-actin. The cDNA was amplified with the following primers.

Mcl-1

forward 5′- AAGCCAATGGGCAGGTCT -3′,

reverse 5′- TGTCCAGTTTCCGAAGCAT -3′

β-actin

forward 5′-CATGTACGTTGCTATCCAGGC-3′,

reverse 5′-CTCCTTAATGTCACGCACGAT-3′

### Transfection of siRNA and plasmid

H82 cells (2 × 10^5^) were seeded onto 6-well plates and incubated for 24 hr. Cells were then transfected with Mcl-1 siRNA and control siRNA (or Mcl-1 expression vector and control vector) using Effectene Transfection Reagent (QIAGEN, Hilden, Germany). Two days after transfection, one part of transfectants was collected for western blot to check whether Mcl-1 was knocked down or over-expressed. Other part of transfectants was replated onto 96-well plate and was treated with DMSO or drugs for 72 hr, and then cell viability was measured by CellTiter-Glo Luminescent assay.

### Xenograft experiments

Animal experiments were carried out according to a protocol approved by Institutional Animal Care and Use Committee of Hefei Institutes of Physical Science. Athymic nude mice were injected subcutaneously in dorsal flank, with a 100 μL suspension of 2 × 10^6^ H526 cells in an equal volume of Matrigel (BD Biosciences, Franklin, NJ, USA). When tumors grew to 4 to 5 mm in diameter, the mice were treated by intraperitonel injection with DMSO control, BEZ235 (2.5 mg/kg), RAD001 (2.5 mg/kg) and the combination of BEZ235 (2.5 mg/kg) with RAD001 (2.5 mg/kg) every 3 days. The tumor size was monitored by caliper measurements and calculated by the formula: Volume = (length × width × width)/2.

### H&E staining and immunohistochemistry

Tumors were harvested from euthanized mice, fixed in 4% paraformaldehyde for 24 hr and embedded in paraffin wax. After sections were cut in 6 μm, sections were dewaxed, rehydrated and stained by Mayer’s hematoxylin and eosin Y solution. Subsequently, the sections were dehydrated and mounted.

For immunohistochemistry, sections were treated in citrate buffer for antigen retrieval. After blocked with 5% BSA, sections were incubated with Ki-67 antibody, secondary antibody, and then stained with DAB (ZSGB-BIO, Beijing, China). Finally, sections were counterstained with hematoxylin, and dehydrated, and then mounted.

### Genomic analysis

By mining the data of 52 SCLC cell lines in the CCLE database, the genetic alterations (mutation status and copy number change) as well as mRNA expression levels (z-score threshold ± 2) of the key molecules in the PI3K/AKT/mTOR pathway were analyzed. The pathway components include: *AKT1*, *AKT2*, *AKT3*, *PIK3CA*, *PIK3CG*, *PIK3R1*, *PIK3R2*, *PTEN*, *MTOR*, *RPTOR*, *RICTOR*, *TSC1* and *TSC2*. OncoPrinter and MutationMapper were generated with the cBioPortal online tool [27, 28].

### Assessment of combination drug synergy

Combination drug synergy was assessed quantitatively using the combination index (CI) method described by Chou [29]. The CI was calculated as CI = (D1/(Dx)1)+(D2/(Dx)2). ‘X’ represents the level of growth inhibition when the two drugs (D1 = dose of drug 1 and D2 = dose of drug 2) combined, while (Dx)1 represents the dose of drug 1 alone required to reach inhibition ‘x’ and (Dx)2 is the dose of drug 2 alone required to reach inhibition ‘x’. (Dx)1 and (Dx)2 were obtained from their individual dose–response curves. In general, “CI < 1” indicates synergism, whereas “CI > 1” indicates antagonism. In addition, “CI < 0.1” indicates very strong synergism, “CI = 0.1–0.3” indicates strong synergism, “CI = 0.3–0.7” indicates synergism, “CI = 0.7–0.85” indicates moderate synergism, “CI = 0.85–0.9” indicates slight synergism.

### Statistical analysis

All data were analyzed using PRISM4 Software (GraphPad Software, Inc., La Jolla, CA, USA). Statistical analysis was performed using an unpaired *t*-test. Results were considered as statistically significant when *P* < 0.05. Correlation analysis between p-4E-BP1 level and SCLC sensitivity to drugs (RAD001 and BEZ235) was performed using the Pearson correlation coefficient.

## SUPPLEMENTARY MATERIALS FIGURES



## Abbreviations

(SCLC): Small cell lung cancer
(NSCLC): Non-small cell lung cancer
(mTORC1): mTOR complex 1
(mTORC2): mTOR complex 2
(CCLE): Cancer Cell Line Encyclopedia
(CI): Combination index

## References

[1] Bunn PA, Minna JD, Augustyn A, Gazdar AF, Ouadah Y, Krasnow MA, Berns A, Brambilla E, Rekhtman N, Massion PP, Niederst M, Peifer M, Yokota J (2016). Small Cell Lung Cancer: Can Recent Advances in Biology and Molecular Biology Be Translated into Improved Outcomes?. J Thorac Oncol.

[2] Hann CL, Rudin CM (2008). Management of small-cell lung cancer: incremental changes but hope for the future. Oncology (Williston Park).

[3] Koinis F, Kotsakis A, Georgoulias V (2016). Small cell lung cancer (SCLC): no treatment advances in recent years. Transl Lung Cancer Res.

[4] Alvarado-Luna G, Morales-Espinosa D (2016). Treatment for small cell lung cancer, where are we now?-a review. Transl Lung Cancer Res.

[5] Umemura S, Mimaki S, Makinoshima H, Tada S, Ishii G, Ohmatsu H, Niho S, Yoh K, Matsumoto S, Takahashi A, Morise M, Nakamura Y, Ochiai A (2014). Therapeutic priority of the PI3K/AKT/mTOR pathway in small cell lung cancers as revealed by a comprehensive genomic analysis. J Thorac Oncol.

[6] Marinov M, Ziogas A, Pardo OE, Tan LT, Dhillon T, Mauri FA, Lane HA, Lemoine NR, Zangemeister-Wittke U, Seckl MJ, Arcaro A (2009). AKT/mTOR pathway activation and BCL-2 family proteins modulate the sensitivity of human small cell lung cancer cells to RAD001. Clin Cancer Res.

[7] Tarhini A, Kotsakis A, Gooding W, Shuai Y, Petro D, Friedland D, Belani CP, Dacic S, Argiris A (2010). Phase II study of everolimus (RAD001) in previously treated small cell lung cancer. Clin Cancer Res.

[8] Strimpakos AS, Karapanagiotou EM, Saif MW, Syrigos KN (2009). The role of mTOR in the management of solid tumors: an overview. Cancer Treat Rev.

[9] Choo AY, Yoon SO, Kim SG, Roux PP, Blenis J (2008). Rapamycin differentially inhibits S6Ks and 4E-BP1 to mediate cell-type-specific repression of mRNA translation. Proc Natl Acad Sci U S A.

[10] Zang C, Eucker J, Liu H, Muller A, Possinger K, Scholz CW (2013). Concurrent inhibition of PI3-kinase and mTOR induces cell death in diffuse large B cell lymphomas, a mechanism involving down regulation of Mcl-1. Cancer Lett.

[11] O’Reilly KE, Rojo F, She QB, Solit D, Mills GB, Smith D, Lane H, Hofmann F, Hicklin DJ, Ludwig DL, Baselga J, Rosen N (2006). mTOR inhibition induces upstream receptor tyrosine kinase signaling and activates Akt. Cancer Res.

[12] Wan X, Harkavy B, Shen N, Grohar P, Helman LJ (2007). Rapamycin induces feedback activation of Akt signaling through an IGF-1R-dependent mechanism. Oncogene.

[13] Dowling RJ, Topisirovic I, Fonseca BD, Sonenberg N (2010). Dissecting the role of mTOR: lessons from mTOR inhibitors. Biochim Biophys Acta.

[14] Yang S, Xiao X, Meng X, Leslie KK (2011). A mechanism for synergy with combined mTOR and PI3 kinase inhibitors. PLoS One.

[15] Nyfeler B, Chen Y, Li X, Pinzon-Ortiz M, Wang Z, Reddy A, Pradhan E, Das R, Lehar J, Schlegel R, Finan PM, Cao ZA, Murphy LO (2012). RAD001 enhances the potency of BEZ235 to inhibit mTOR signaling and tumor growth. PLoS One.

[16] Ren H, Chen M, Yue P, Tao H, Owonikoko TK, Ramalingam SS, Khuri FR, Sun SY (2012). The combination of RAD001 and NVP-BKM120 synergistically inhibits the growth of lung cancer *in vitro* and *in vivo*. Cancer Lett.

[17] Passacantilli I, Capurso G, Archibugi L, Calabretta S, Caldarola S, Loreni F, Delle Fave G, Sette C (2014). Combined therapy with RAD001 e BEZ235 overcomes resistance of PET immortalized cell lines to mTOR inhibition. Oncotarget.

[18] Barretina J, Caponigro G, Stransky N, Venkatesan K, Margolin AA, Kim S, Wilson CJ, Lehar J, Kryukov GV, Sonkin D, Reddy A, Liu M, Murray L (2012). The Cancer Cell Line Encyclopedia enables predictive modelling of anticancer drug sensitivity. Nature.

[19] Ducker GS, Atreya CE, Simko JP, Hom YK, Matli MR, Benes CH, Hann B, Nakakura EK, Bergsland EK, Donner DB, Settleman J, Shokat KM, Warren RS (2014). Incomplete inhibition of phosphorylation of 4E-BP1 as a mechanism of primary resistance to ATP-competitive mTOR inhibitors. Oncogene.

[20] Musa J, Orth MF, Dallmayer M, Baldauf M, Pardo C, Rotblat B, Kirchner T, Leprivier G, Grunewald TG (2016). Eukaryotic initiation factor 4E-binding protein 1 (4E-BP1): a master regulator of mRNA translation involved in tumorigenesis. Oncogene.

[21] Guertin DA, Sabatini DM (2009). The pharmacology of mTOR inhibition. Sci Signal.

[22] Maira SM, Stauffer F, Brueggen J, Furet P, Schnell C, Fritsch C, Brachmann S, Chene P, De Pover A, Schoemaker K, Fabbro D, Gabriel D, Simonen M (2008). Identification and characterization of NVP-BEZ235, a new orally available dual phosphatidylinositol 3-kinase/mammalian target of rapamycin inhibitor with potent *in vivo* antitumor activity. Mol Cancer Ther.

[23] Nishikawa M, Miyake H, Behnsawy HM, Fujisawa M (2015). Significance of 4E-binding protein 1 as a therapeutic target for invasive urothelial carcinoma of the bladder. Urol Oncol.

[24] Nishi T, Iwasaki K, Ohashi N, Tanaka C, Kobayashi D, Nakayama G, Koike M, Fujiwara M, Kobayashi T, Kodera Y (2013). Phosphorylation of 4E-BP1 predicts sensitivity to everolimus in gastric cancer cells. Cancer Lett.

[25] Darb-Esfahani S, Faggad A, Noske A, Weichert W, Buckendahl AC, Muller B, Budczies J, Roske A, Dietel M, Denkert C (2009). Phospho-mTOR and phospho-4EBP1 in endometrial adenocarcinoma: association with stage and grade *in vivo* and link with response to rapamycin treatment *in vitro*. J Cancer Res Clin Oncol.

[26] Lui VW, Thomas SM, Zhang Q, Wentzel AL, Siegfried JM, Li JY, Grandis JR (2003). Mitogenic effects of gastrin-releasing peptide in head and neck squamous cancer cells are mediated by activation of the epidermal growth factor receptor. Oncogene.

[27] Gao J, Aksoy BA, Dogrusoz U, Dresdner G, Gross B, Sumer SO, Sun Y, Jacobsen A, Sinha R, Larsson E, Cerami E, Sander C, Schultz N (2013). Integrative analysis of complex cancer genomics and clinical profiles using the cBioPortal. Sci Signal.

[28] Cerami E, Gao J, Dogrusoz U, Gross BE, Sumer SO, Aksoy BA, Jacobsen A, Byrne CJ, Heuer ML, Larsson E, Antipin Y, Reva B, Goldberg AP (2012). The cBio cancer genomics portal: an open platform for exploring multidimensional cancer genomics data. Cancer Discov.

[29] Chou TC (2006). Theoretical basis, experimental design, and computerized simulation of synergism and antagonism in drug combination studies. Pharmacol Rev.

