# Supplementary material for: Combination treatment of RAD001 and BEZ235 exhibits synergistic antitumor activity via down-regulation of p-4E-BP1/Mcl-1 in small cell lung cancer
Source: Oncotarget. 2017 Jul 4;8(63):106486–98. doi: 10.18632/oncotarget.18984 (PMC5739750; doi:10.18632/oncotarget.18984)
Supplement: Supplementary file 1 [file oncotarget-08-106486-s001.pdf]

Combination treatment of RAD001 and BEZ235 exhibits synergistic antitumor activity via down-regulation of p-4E-BP1/Mcl-1 in small cell lung cancer

SUPPLEMENTARY MATERIALS

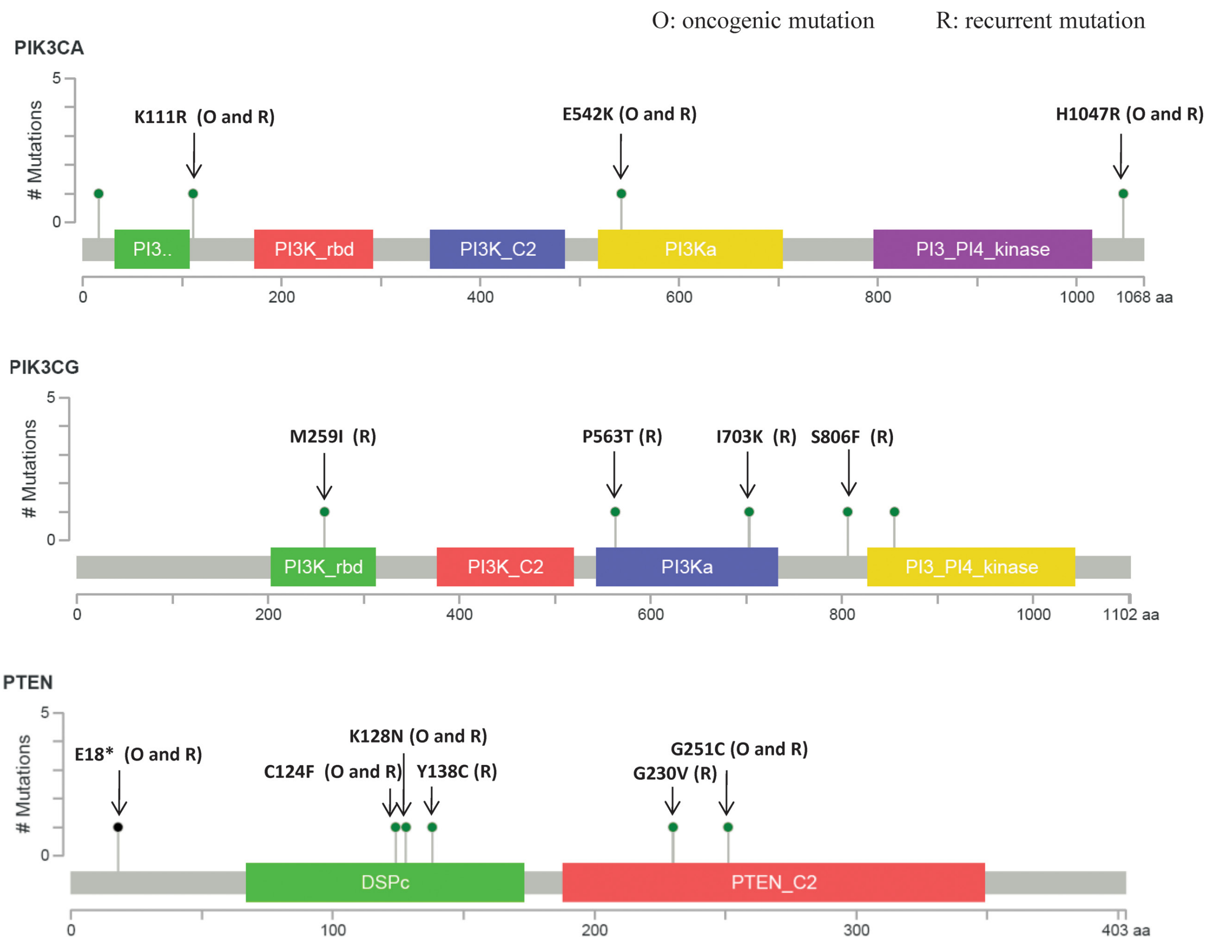

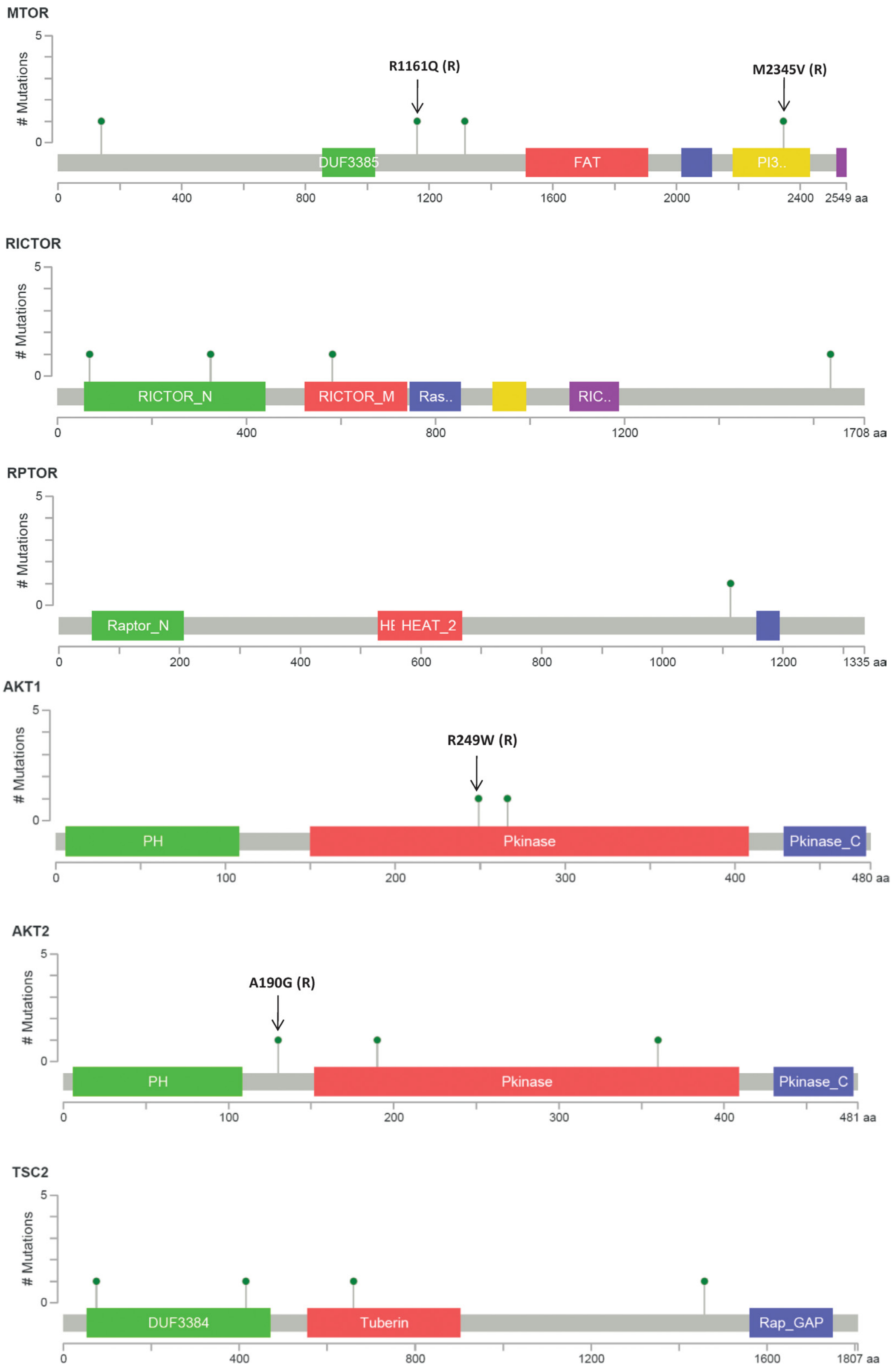

Supplementary Figure 1: Nine mutated genes in PI3K/AKT/mTOR pathway identified among SCLC cell lines. The position of mutations is displayed relative to known functional domains of the encoded proteins. Each mutation is represented by a cycle: missense mutations (green circles); nonsense mutations (black circle). Mutation data were generated by CCLE and obtained via the cBioPortal for Cancer Genomics (URL: http://www.cbioportal.org/public-portal/).

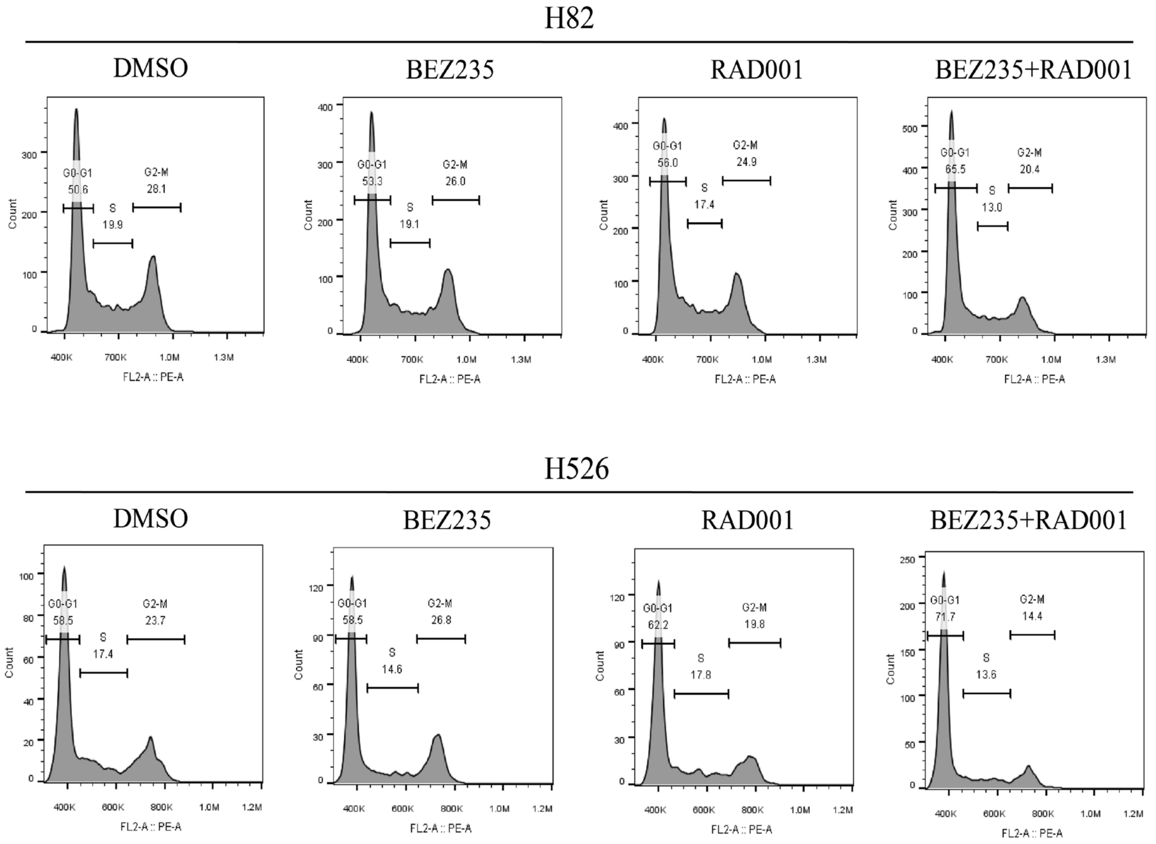

Supplementary Figure 2: Original result of cell cycle analyses by flow cytometry for BEZ235 and RAD001 single and combination treatment in H82 and H526 cells.

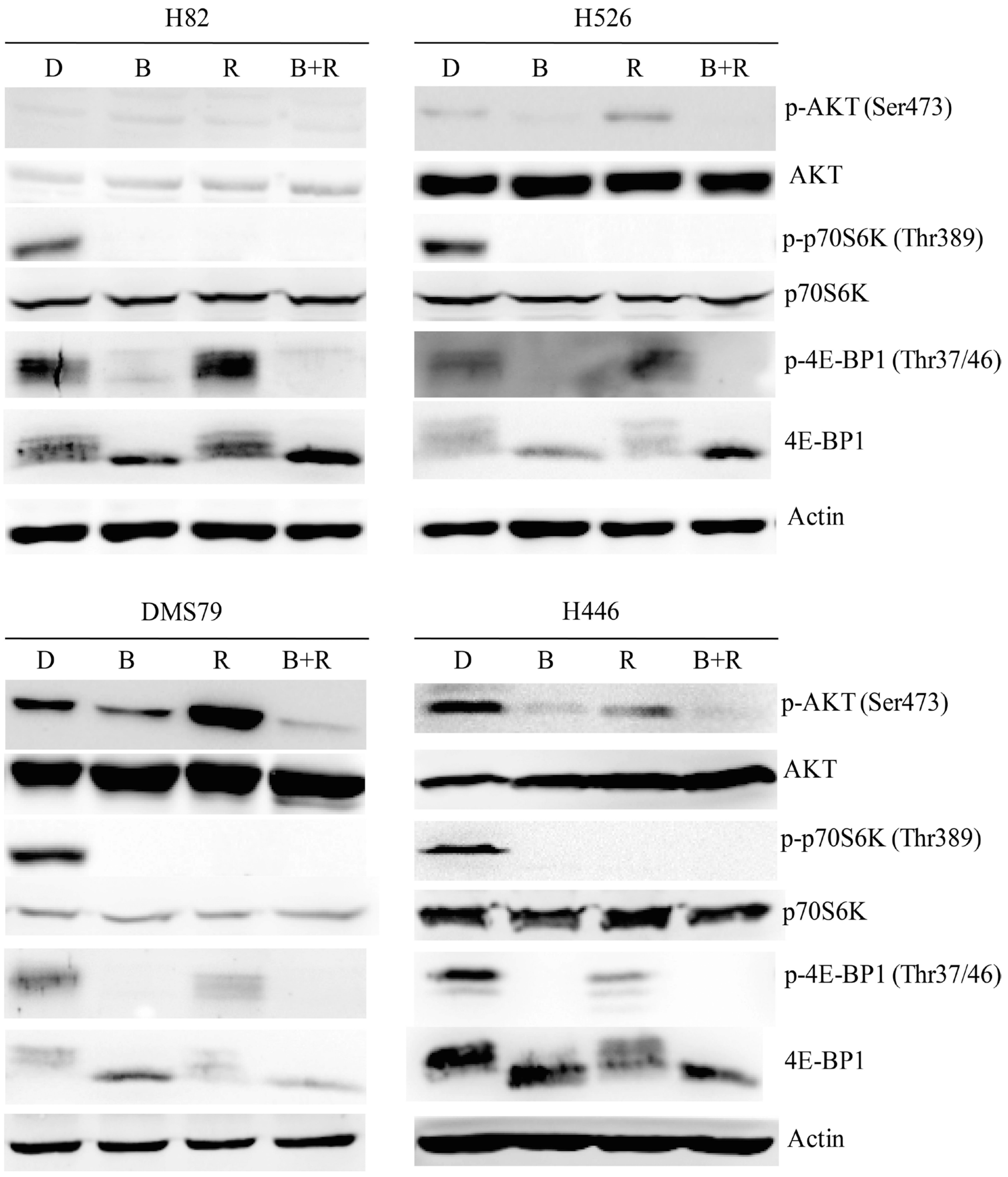

Supplementary Figure 3: Remarkable inhibition of PI3K/AKT/mTOR signaling of SCLC cells by RAD001 and BEZ235 co-treatment. SCLC cells (H82, H526, DMS79 and H446) were treated with DMSO control, RAD001 (100 nM), BEZ235 (100 nM) or the combination of RAD001 (100 nM) and BEZ235 (100 nM) for 12 hr. Cellular proteins were collected for western blot. D: DMSO, B: BEZ235, R: RAD001, B+R: BEZ235+RAD001.
